# Supplementary material for: A Global Screen for Assembly State Changes of the Mitotic Proteome by SEC-SWATH-MS
Source: Cell Syst. 2020 Feb 26;10(2):133–155.e6. doi: 10.1016/j.cels.2020.01.001 (PMC7042714; doi:10.1016/j.cels.2020.01.001)

Q9ULJ6 | ZMIZ1_HUMAN | ZMIZ1 KIAA1224 RAI17 ZIMP10
Monomer MW [kDa]: 115.483 Monomer expected elution fraction: 39

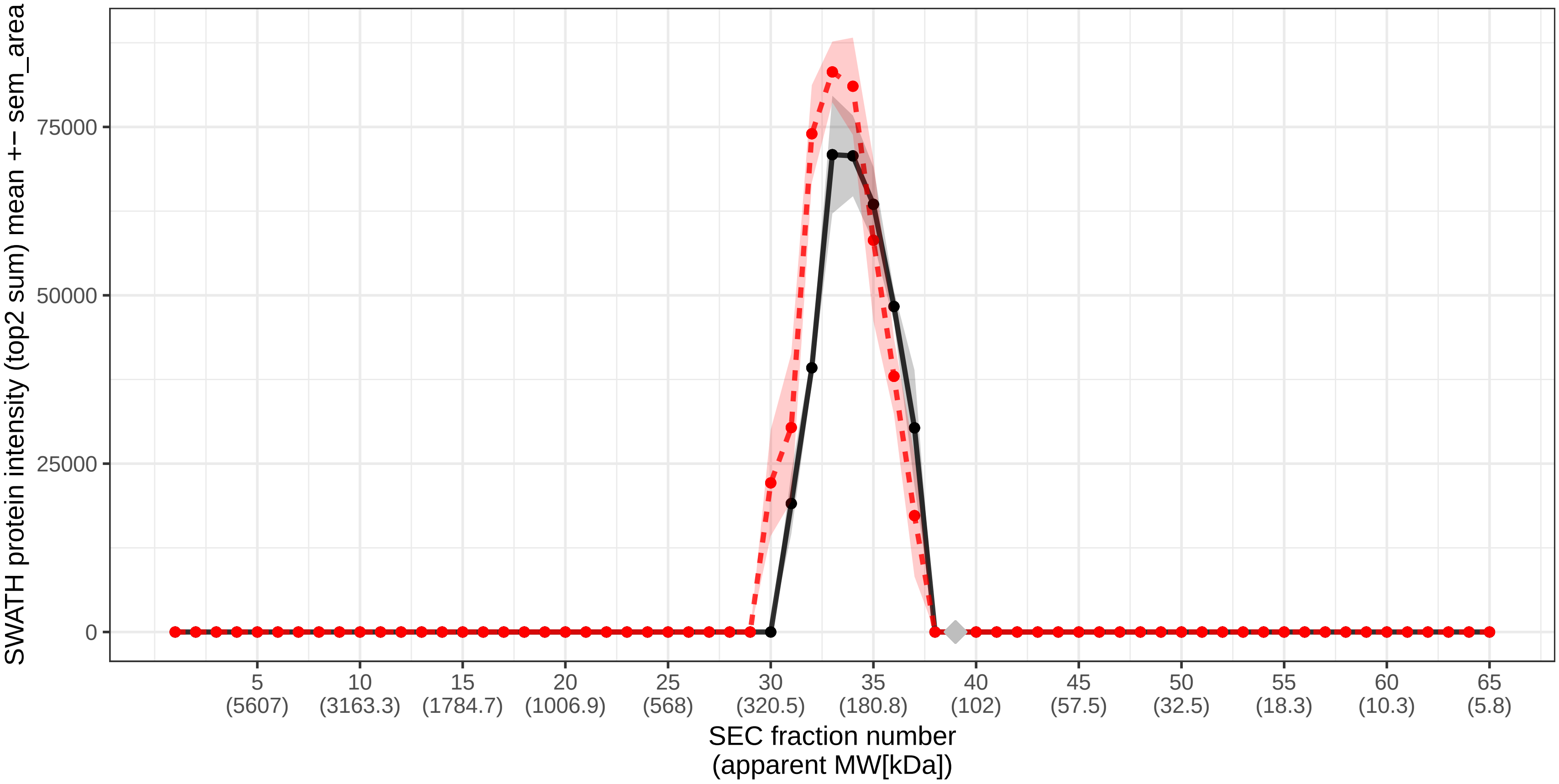

Supplement: Data S1. SEC-SWATH-MS Protein Chromatograms, Related to Figure 1 [file mmc6.zip › SECchrom_Q9ULJ6_ZMIZ1_HUMAN_ZMIZ1_KIAA1224_RAI17_ZIMP10.pdf]
